# From Pollen to Plate: A Botanical Classification-Based Narrative Review of Fresh Fruit Allergy

**DOI:** 10.3390/nu18203377

**Published:** 2026-10-15

**Authors:** Luca Pecoraro, Carla Mastrorilli, Riccardo Castagnoli, Francesca Mori, Stefania Arasi, Angela Klain, Lucia Liotti, Simona Barni, Mariannita Gelsomino, Mattia Giovannini, Lucia Caminiti, Cristiana Indolfi, Francesca Saretta, Gian Luigi Marseglia, Elio Novembre

**Affiliations:** 1 Department of Experimental Medicine, Pediatric Section, University of Salento, Hospital “Vito Fazzi”, 73100 Lecce, Italy; 2 Pediatric Hospital Giovanni XXIII, Pediatric and Emergency Department, AOU Policlinic of Bari, 70100 Bari, Italy; 3 Pediatric Clinic, Fondazione IRCCS Policlinico San Matteo, 27100 Pavia, Italy; 4 Allergy Unit, Meyer Children’s Hospital IRCCS, 50139 Florence, Italy; 5 Allergy Diseases Research Area, Pediatric Allergology Unit, Bambino Gesù Children’s Hospital, IRCCS, 00146 Rome, Italy; 6 Department of Woman, Child and General and Specialized Surgery, University of Campania Luigi Vanvitelli, 80100 Naples, Italy; 7 Pediatric Unit, Department of Mother and Child Health, Salesi Children’s Hospital, 60123 Ancona, Italy; 8 Pediatric Allergy Unit, Department of Life Sciences and Public Health, Fondazione Policlinico Universitario A. Gemelli IRCCS, Catholic University of the Sacred Heart, 00168 Rome, Italy; 9 Department of Health Sciences, University of Florence, 50139 Florence, Italy; 10 Department of Human Pathology in Adult and Development Age “Gaetano Barresi”, Allergy Unit, Department of Pediatrics, AOU Policlinico Gaetano Martino, 98100 Messina, Italy; 11 Pediatric Department, Latisana-Palmanova Hospital, Azienda Sanitaria Universitaria Friuli Centrale, 33100 Udine, Italy

**Keywords:** fruit allergy, botanical classification, cross-reactivity, component-resolved diagnostics, skin prick test, specific IgE, oral food challenge, lipid transfer proteins, gibberellin-regulated proteins, oral allergy syndrome, anaphylaxis

## Abstract

Fruit consumption is a cornerstone of a healthy diet, providing essential micronutrients and bioactive compounds; however, fresh fruits may also trigger IgE-mediated allergic reactions, ranging from mild oral symptoms to potentially life-threatening anaphylaxis. Fruit allergy is a heterogeneous field characterized by diverse clinical phenotypes, botanical relationships, molecular sensitization profiles, and patterns of cross-reactivity. This narrative review organizes the available evidence according to 18 botanical families, adapting and extending an established botanical classification framework to the specific context of fresh fruit allergy. Botanical classification serves as a structured organizational framework rather than as a predictor of allergenic cross-reactivity, which is primarily determined by shared or homologous allergenic proteins and may occur both within and across botanical families. The review integrates botanical relationships with clinical manifestations, molecular allergen families, cross-reactivity, cofactors, diagnosis, and management. Clinical assessment should be individualized and based on the clinical history and appropriate in vivo and in vitro testing, including skin prick or prick-to-prick testing with fresh fruit and measurement of specific IgE, with oral food challenges reserved for selected cases. Molecular allergology may provide additional information for risk assessment in selected patients, although the availability and strength of evidence for individual molecular components vary considerably. Management should likewise be individualized according to the confirmed trigger, clinical phenotype, and risk of systemic reactions, with appropriate dietary counselling and patient education. The available epidemiological and clinical evidence is heterogeneous, particularly for uncommon fruits and in pediatric populations. It should therefore be interpreted in the context of study design, geographic setting, and diagnostic approach. Integrating botanical classification with molecular allergology and clinical phenotyping may provide a useful framework for organizing the fragmented evidence on fresh fruit allergy and informing individualized diagnostic and management approaches.

## 1. Introduction

Regular consumption of fruits plays a central role in human nutrition, as they are key sources of essential micronutrients and bioactive compounds that support overall health and may influence the risk of chronic diseases [1]. Owing to the wide diversity of fruits available worldwide, substantial variability exists in their nutritional composition and phytochemical profiles. This heterogeneity has important implications not only for dietary assessment and nutritional guidance but also for the understanding of adverse immune responses, including food allergy [2]. From a research perspective, the classification of fruits is a critical methodological step when exploring relationships between diet and health outcomes. Accurate categorization enables investigators to assess exposure to specific food components meaningfully and to design dietary assessment tools that capture relevant intake patterns [3].

While classification systems have traditionally been developed for nutritional or public health purposes, they also provide a useful framework for allergological research. In the context of fruit allergy, grouping fruits by shared characteristics may help identify patterns of sensitization, cross-reactivity, and clinical presentation. However, much of the existing literature addresses fruit allergy in a broad age range, often extrapolating findings from adult populations [2]. Pediatric-specific data remain comparatively limited, even though immune maturation, dietary exposure, and clinical expression of food allergy differ substantially in children [4]. Where available, prevalence estimates should be interpreted cautiously, as reported sensitization or pollen-associated cross-reactivity does not necessarily indicate clinically confirmed fruit allergy. The present article is a narrative review of fruit allergy. It adopts an established fruit classification framework described by Pennington [2], which categorizes fruits according to botanical families. This framework served as the starting point for the present review and was subsequently adapted based on the allergological literature. Specifically, additional botanical families were incorporated when published evidence of allergy to fresh fruits from those families was identified, even when those fruits were not represented in the original classification. The resulting framework was therefore developed to comprehensively organize the available evidence on fresh fruit allergy rather than to reproduce a fixed or exhaustive botanical classification of all edible fruits. In the present review, this botanical framework is reinterpreted from an allergological perspective, with particular emphasis on the clinical phenotypes, molecular sensitization profiles, cross-reactivity patterns, diagnostic challenges, and management implications observed in children and adults. This approach allows the botanical relationships among fruits to be considered together with the molecular and clinical mechanisms underlying fruit allergy, including sensitization to different allergen families and cross-reactivity occurring both within and across botanical groups. According to this adapted framework, the fruits included in the review are distributed across 18 botanical families. Several families are represented by a single fruit, such as kiwifruit (Actinidiaceae), mango (Anacardiaceae), pineapple (Bromeliaceae), papaya (Caricaceae), banana (Musaceae), fig (Moraceae), and pomegranate (Lythraceae). Other families include multiple fruit members and are therefore of particular interest when considering shared allergenic potential. The Cucurbitaceae family comprises various melons, including cantaloupe, casaba, honeydew, and watermelon. The Ericaceae family includes berries such as blueberries and cranberries. The Arecaceae family includes date and coconut. The Rosaceae family is the largest, encompassing a wide range of commonly consumed fruits, including apples, pears, stone fruits, and several berries. Similarly, the Rutaceae family includes multiple citrus fruits, such as oranges, lemons, limes, and grapefruits, as well as related varieties [2]. The Vitaceae family includes grapes and raisins. Lauraceae, Ebenaceae, Myricaceae, Cactaceae, and Sapindaceae may also be considered within this classification (Table 1) [5].

Against this background, the present review aims to organize the heterogeneous evidence on fresh fruit allergy according to these 18 botanical families, while integrating botanical relationships with clinical phenotypes, molecular allergen profiles, cross-reactivity, diagnostic approaches, and management. The strength of the available evidence varies considerably among fruit families and specific cross-reactivity patterns. Well-characterized associations are supported by multiple studies, whereas evidence for some uncommon fruits is limited to small series, individual case reports, or isolated molecular observations and should therefore be interpreted cautiously. The rationale for adopting this framework is therefore not to infer clinical cross-reactivity from botanical relatedness, but to provide a consistent and clinically oriented structure for bringing together fragmented evidence across commonly consumed and less frequently reported fruits. By linking each botanical family to the available allergological evidence, this approach may facilitate the interpretation of molecular sensitization patterns and clinical phenotypes while highlighting areas in which evidence remains limited.

## 2. Methods

**Figure 1:**
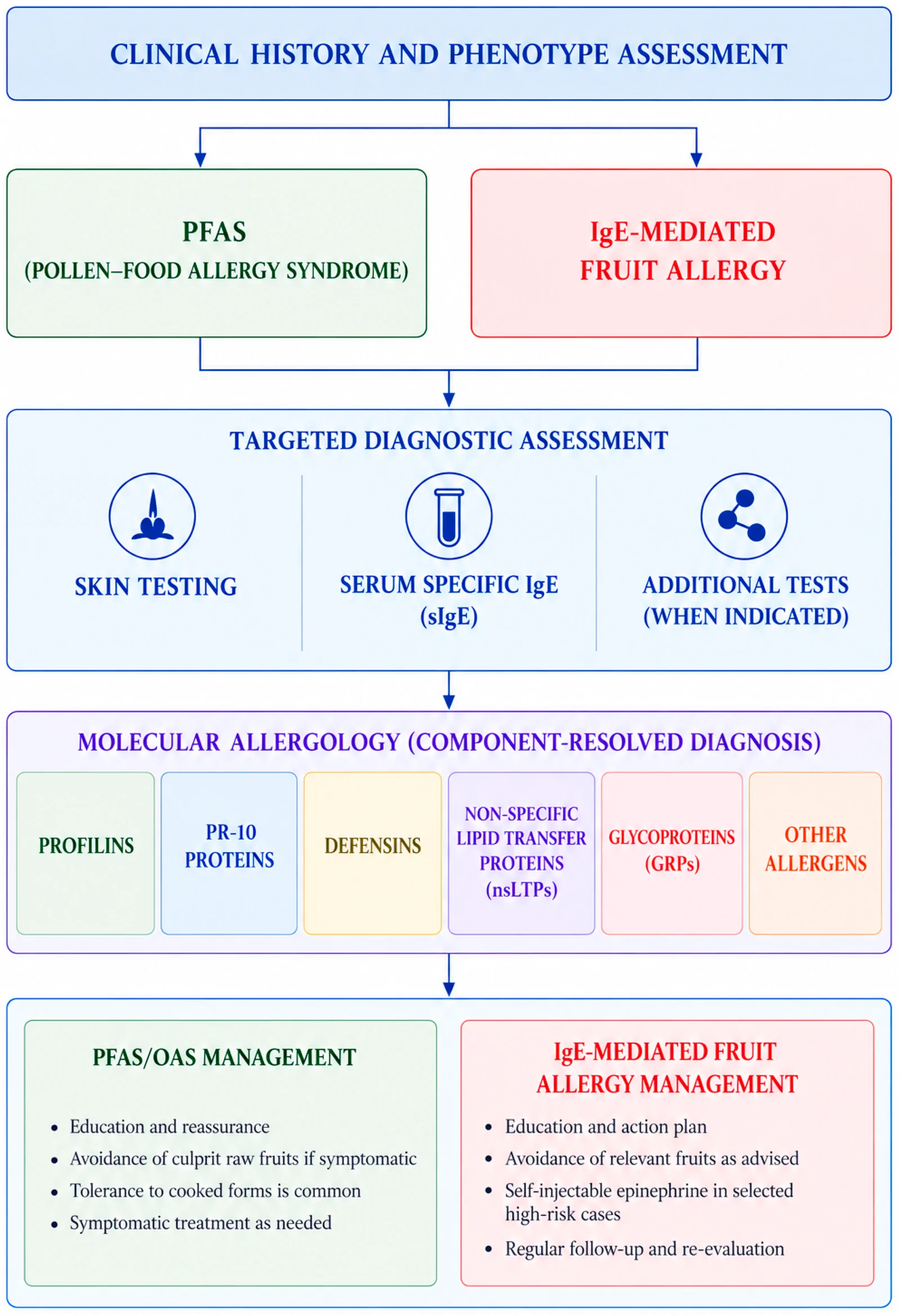
Proposed clinical pathway for the assessment and management of fresh fruit allergy.

A structured literature search was performed using the PubMed/MEDLINE database, supplemented by manual screening of reference lists of relevant publications. Publications from January 1980 to August 2025 were considered. The search strategy used combinations of Medical Subject Headings (MeSH) terms and free-text keywords related to fruit allergy, individual fruit families, clinical phenotypes, pollen-food allergy syndrome, anaphylaxis, cross-reactivity, component-resolved diagnostics, children, and individual fruit species, including “fruit allergy”, “fresh fruit allergy”, “food allergy”, “oral allergy syndrome”, “pollen-food allergy syndrome”, “anaphylaxis”, “cross-reactivity”, “component-resolved diagnostics”, “children”, “pediatric”, “kiwifruit”, “apple”, “peach”, “citrus”, “banana”, “avocado”, “persimmon”, “dragon fruit”, “papaya”, “pineapple”, “grape”, and “pomegranate”. The search retrieved approximately 3500 records. The literature was considered according to its relevance to the review’s objectives, with particular attention to study design, methodological quality, and its contribution to understanding epidemiology, clinical manifestations, allergenic components, cross-reactivity, diagnosis, and management of fresh fruit allergy. The review considered original studies, cohort and case–control studies, case series, case reports, randomized controlled trials, systematic reviews, and narrative reviews published in English. Particular attention was given to evidence from pediatric populations whenever available. For uncommon fruit allergies, conference abstracts were considered when they provided relevant information not available in full-text publications; these were used only as supplementary evidence and were clearly identified as such in the reference list. Editorials, commentaries, letters without original data, and animal studies were not considered. Given the narrative nature of the review, no formal systematic-review protocol, predefined systematic selection workflow, or quantitative synthesis was applied. The final body of evidence was selected to provide a clinically oriented and comprehensive overview of the available literature, while recognizing that the amount and strength of evidence vary substantially among individual fruits and botanical families. During the preparation of this manuscript, the authors used ChatGPT (GPT-5.6 Luna; OpenAI, San Francisco, CA, USA) to assist with the generation and graphical design of the Graphical Abstract and Figure 1. The authors reviewed and edited the generated material and take full responsibility for the scientific content and final version of the figures.

## 3. Botanical Families

### 3.1. Actinidiaceae

The Actinidiaceae family encompasses commercial kiwifruit (*Actinidia deliciosa*) and its hardy varieties. Reported prevalence estimates for kiwifruit allergy vary substantially according to the study population and diagnostic approach. In a population-based birth cohort from the United Kingdom, the prevalence of kiwifruit allergy during childhood was 0.1–0.2%. Much higher proportions have been reported in selected clinical cohorts of children with established fruit and vegetable allergy and should not be extrapolated to the general pediatric population [6]. Kiwifruit allergy can occur across all age groups but is frequently reported in children [7]. Children appear to exhibit a distinct clinical phenotype compared with adults, being more frequently monosensitized to kiwifruit and less commonly affected by pollen-associated cross-reactivity; primary gastrointestinal sensitization has been proposed as a possible mechanism in pediatric patients [7]. Clinically, reactions range from mild oral symptoms to life-threatening anaphylaxis [8]. Oral allergy syndrome (OAS), characterized by rapid-onset itching, tingling, and swelling of the lips, throat, and tongue, is common and may occur in association with pollen allergy through cross-reactivity between homologous allergens [9]. However, systemic reactions, including urticaria, abdominal pain, vomiting, respiratory distress, and anaphylaxis, have also been reported [10,11]. Severe systemic reactions and anaphylaxis appear to occur more frequently in children than in adults [7], while food-dependent exercise-induced anaphylaxis (FDEIA) represents a particularly relevant clinical phenotype [11].

The allergenic profile of kiwifruit is complex and includes Act d 1 (actinidin), Act d 2 (a thaumatin-like protein), Act d 5 (a kiwellin), and Act d 8 (a pathogenesis-related protein, PR-10) [9,12]. Commercial kiwifruit includes both green kiwifruit (*Actinidia deliciosa*) and gold kiwifruit (*Actinidia chinensis*), which show partially distinct allergen profiles. Gold kiwifruit contains homologous allergens, including Act c 8 (PR-10) and Act c 10 (non-specific lipid transfer protein, nsLTP), in addition to Act c 5 (kiwellin) [12]. Act d 8 is homologous to the birch pollen allergen Bet v 1 and is therefore particularly relevant to pollen-food syndrome [9,12]. Its heat and digestive lability may contribute to the predominantly localized oropharyngeal symptoms observed in patients sensitized to PR-10. In contrast, more thermostable allergens such as Act d 1 and Act d 2 are more frequently associated with systemic reactions [10,12]. The coexistence of PR-10 proteins, profilins, lipid transfer proteins (LTPs), and seed storage proteins further contributes to the complex patterns of molecular sensitization and cross-reactivity observed in kiwifruit allergy. Diagnosis may be challenging because different molecular sensitization profiles can underlie apparently similar clinical presentations. Although clinical history, skin prick testing (SPT), and measurement of specific IgE (sIgE) remain central to diagnosis [7,10], whole-kiwi extract-based SPT and sIgE may yield false-negative results, particularly in patients reactive to Act d 8 [10]. Component-resolved diagnostics (CRD), by assessing sIgE to individual purified or recombinant allergens, may therefore provide additional information for molecular characterization and risk assessment [10,11,12]. In children, the absence of sensitization to Act d 8 has been associated with an increased likelihood of systemic allergic reactions, suggesting a potential role for molecular diagnostics in pediatric risk stratification [7].

Therapeutic evidence remains limited. Mempel et al. investigated sublingual immunotherapy (SLIT) using a non-standardized extract prepared from homogenized fresh kiwi pulp, serially diluted and administered sublingually with gradual dose escalation until daily tolerance to fresh kiwi was achieved. Short-term treatment induced clinical desensitization despite mild dose-related reactions, while long-term follow-up showed sustained tolerance [13].

### 3.2. Anacardiaceae

The fruit of the Anacardiaceae family is mainly represented by mango (*Mangifera indica*). Mango allergy is a clinically significant condition with a broad spectrum of manifestations, ranging from localized contact dermatitis to potentially life-threatening anaphylaxis. Miell et al. reported the first anaphylactic reaction following mango ingestion [14]. Clinical phenotypes include both IgE-mediated and non-IgE-mediated reactions, with the latter often associated with contact with the fruit peel. IgE-mediated reactions may manifest as OAS or severe systemic reactions. Sareen et al. described acute exacerbation of asthma following mango ingestion [15], while Ukleja-Sokołowska et al. and Ortega-Martín et al. reported cases of anaphylaxis following mango consumption [16,17]. Contact dermatitis may also occur, particularly following exposure to the peel. Mango peel contains urushiol, an allergenic compound also found in poison ivy, which can induce delayed-type hypersensitivity and allergic contact dermatitis, including among individuals who handle the fruit without ingesting it [18,19].

From a molecular perspective, mango contains several characterized allergens. Early work, including the review by Hassan and Venkatesh, described potential allergenic proteins, while subsequent studies have provided more precise molecular characterization [20]. Man i 1 (class IV chitinase), Man i 2 (pathogenesis-related protein), Man i 4 (profilin), and a panallergen cross-reacting with the mugwort defensin Art v 1 have been identified and may support CRD of mango allergy [16,21,22]. Mango allergy also displays complex patterns of cross-reactivity. Protein homology may result in sensitization to other Anacardiaceae members, including cashew and pistachio; however, van der Valk et al. reported a relatively low rate of clinically relevant cosensitization between cashew and mango in children, indicating that positive IgE results do not necessarily predict clinical reactivity [23]. In addition, mango allergy may be associated with pollen-food cross-reactivity mediated by profilins and LTPs, with shared or homologous allergens identified in birch, mugwort, and peach [20,21]. Epidemiological data on mango allergy remain limited. A population-based study by Li et al. on pediatric fruit and vegetable allergy provides a broader epidemiological context, including reactions to mango [24]. Diagnosis relies on clinical history, SPT, serum-specific IgE testing, and, when appropriate, CRD [21]. This molecular characterization may improve risk assessment and guide management. It should be individualized according to the clinical phenotype and allergenic profile. In patients with predominantly local reactions to heat-labile allergens, avoidance of raw fruit and tolerance of cooked or processed forms may be considered based on individual history. In contrast, more stable allergens such as LTPs may retain their allergenic activity after processing, and processed products may therefore still pose a risk [25].

Systemic reactions are less common but may occur, particularly in patients with polysensitization or in the presence of cofactors such as exercise, alcohol, fever, or NSAID use. Adrenaline/epinephrine auto-injectors should be considered in selected patients with previous systemic reactions or an increased individual risk of anaphylaxis [25]. Future research includes developing improved methods for detecting mango allergens in processed foods, such as immunomagnetic nanoparticle-enhanced approaches, which may enhance safety for allergic consumers [26].

### 3.3. Bromeliaceae

Pineapple (*Ananas comosus*) allergy encompasses a spectrum of immunological responses, with both molecular and non-immunologic mechanisms contributing to its clinical manifestations. The major molecular allergen reported for pineapple is Ana c 1, a profilin involved in pollen-food cross-reactivity, particularly in pollen-sensitized individuals [27,28,29,30,31]. Another relevant allergen is Ana c 2 (bromelain), a cysteine protease associated with IgE reactivity and cross-reactive carbohydrate determinants (CCDs) [32,33].

Clinically, OAS is commonly reported, although systemic reactions, including anaphylaxis, have also been described [34,35,36,37]. Beyond its role as an allergen, bromelain has proteolytic activity that can induce non-immunologic mucosal irritation, potentially mimicking IgE-mediated reactions and representing an important differential diagnostic consideration. Diagnostic approaches include SPT, specific IgE measurement, CRD, and oral food challenges (OFC) [29,30,31].

Processing may influence pineapple allergenicity, with thermal treatment potentially reducing the reactivity of some labile allergens while having limited effects on more stable proteins [29]. Thus, patients with predominantly local reactions may sometimes tolerate cooked or processed forms, whereas this approach should not be considered protective in patients with a history of systemic reactions. From a practical perspective, pineapple is also present in beverages, fruit mixtures, and processed foods, making it important to read food labels when avoiding allergens.

### 3.4. Caricaceae

Papaya (*Carica papaya*) allergy is frequently associated with latex-fruit syndrome, in which sensitization to natural rubber latex proteins leads to cross-reactive allergic responses to various fruits, including papaya [38]. This association, known as latex-papaya syndrome, may present with a spectrum of clinical phenotypes ranging from OAS to more severe systemic reactions [39,40].

From a molecular perspective, chymopapain represents a relevant papaya allergen, while Cari p 1, an endopolygalacturonase, has also been identified as an allergen [40,41,42,43]. Molecular testing may help distinguish primary papaya allergy from IgE-mediated cross-reactivity with latex, particularly involving class II chitinases (Car p chitinase), which have been implicated in latex-fruit syndrome [41]. A class II chitinase has been characterized as a 28 kDa protein with structural features consistent with this enzyme family, supporting its role as a relevant cross-reactive allergen in latex-fruit allergy [41]. Diagnostic assessment includes SPT, specific IgE measurement, and, when indicated, OFC. Thermal processing may reduce the reactivity of some labile allergens while having a limited effect on more stable proteins such as chymopapain [40].

### 3.5. Cucurbitaceae

Watermelon (*Citrullus lanatus*) and melon (*Cucumis melo*) are the most representative fruits of the Cucurbitaceae family. Allergic phenotypes are heterogeneous, with cross-reactivity playing an important role. The first report of watermelon allergy dates to the 1980s, when Enberg et al. demonstrated shared allergens between watermelon and ragweed, establishing an important pollen-fruit cross-reactivity pattern [44]. OAS is the most frequently reported phenotype, whereas anaphylaxis appears to be uncommon [45].

Molecular characterization has identified several relevant allergens in watermelon. Profilin has been characterized as a major allergen and has served as a model for plant-derived food profilins, contributing substantially to pollen-fruit cross-reactivity [46,47]. In a study of 53 subjects who reported adverse reactions to melon, Rodriguez et al. provided clinical evidence of allergenicity [48]. The major melon allergen was subsequently identified as the profilin Cuc m 2, which shows significant allergenic reactivity and contributes to cross-reactivity patterns [49]. Its susceptibility to pepsin digestion in patients with OAS may help explain the predominantly oropharyngeal symptoms observed in many cases [50]. In addition, nsLTPs present in Cucurbitaceae peels exhibit cross-reactivity and may be associated with more severe systemic reactions [51,52]. Winter melon has also been reported to cause anaphylaxis through cross-reactivity associated with ragweed pollen sensitization [53]. Diagnosis of watermelon and melon allergy is based on clinical history, SPT, specific IgE measurement, and, when necessary, an OFC. CRD may provide additional information for distinguishing primary fruit allergy from pollen-associated molecular sensitization.

### 3.6. Ericaceae

Blueberries, cranberries, lingonberries, bilberries, and huckleberries belong to the Ericaceae family. Blueberry allergy is rare. Dereci et al. reported an extremely low prevalence of blueberry allergy in a Turkish population undergoing double-blind, placebo-controlled food challenge, estimated at approximately 0.00006% [54]. This estimate is based on a specific study population and should not be interpreted as a population-wide prevalence of blueberry allergy [54]. Clinical manifestations of blueberry allergy may vary, including severe systemic reactions; for example, a Chinese child experienced blueberry-induced anaphylaxis associated with LTP sensitization [55,56]. Cranberry and lingonberry allergy share similar clinical characteristics, while their allergenic potential may be related to their botanical relationship with other Ericaceae fruits.

From a molecular perspective, cross-reactivity within the Ericaceae family has also been reported [57]. LTPs, PR-10 proteins, and profilins may contribute to different patterns of molecular sensitization and pollen–fruit cross-reactivity [56,57,58]. The diagnosis is based on clinical history, SPT, specific IgE measurement, and, when necessary, OFC. CRD may help distinguish LTP and profilin sensitization.

### 3.7. Moraceae

The Moraceae family includes fig (*Ficus carica*), mulberry (*Morus* spp.), breadfruit (*Artocarpus altilis*), jackfruit (*Artocarpus heterophyllus*), and osage orange (*Maclura pomifera*). Fig and mulberry are the most commonly reported triggers of allergic reactions within this botanical family. Antico et al. described OAS to fig in patients whose main allergic manifestations were associated with sensitization to grass and birch pollens [59]. More severe systemic reactions have also been reported. Focke et al. demonstrated cross-reactivity between *Ficus benjamina* latex and fig fruit in patients with clinical fig allergy [60]. Hemmer et al. further investigated cross-reactivity with birch pollen, identified foods associated with the Ficus-fruit syndrome, and established a relationship with natural rubber latex allergy [61,62]. Urbani et al. described a patient with an adverse reaction to *Ficus carica* and possible cross-reactivity with Der p 1, suggesting a potential relationship with house dust mite sensitization [63]. Mulberry allergy is also represented within the Moraceae family [64]. The IgE-mediated nature of mulberry fruit allergy was demonstrated by Choi et al., who reported anaphylaxis following mulberry fruit consumption [65].

Cross-reactivity between fig and mulberry was described by Caiaffa et al., highlighting shared allergenic components within Moraceae fruits [66,67].

Occupational exposure may also be relevant. Jiang et al. reported a case of papain-induced occupational asthma associated with kiwi and fig allergy, emphasizing the importance of considering occupational exposure in patients with fruit allergy [68].

The clinical spectrum of Moraceae fruit allergy, therefore, ranges from OAS to systemic reactions, including anaphylaxis. Diagnosis is based on clinical history, SPT, specific IgE measurement, and, when necessary, OFC. In addition, CRD may help characterize molecular sensitization, including LTP and profilin sensitization.

### 3.8. Musaceae

The Musaceae family comprises flowering plants native to tropical regions of Africa and Asia and includes several genera [69]. The most widespread genus is *Musa*, which includes banana, the family’s best-known fruit. The reported prevalence of banana allergy in the general population is approximately 0.06%, although estimates vary according to the study population and diagnostic approach [70,71,72,73]. Banana sensitization has also been documented in selected clinical populations. In a cohort of respiratory-allergic patients in Calcutta, India, skin prick test reactivity to banana was observed in 40.6% of the 330 subjects tested, whereas banana was identified as a cause of delayed food sensitization by patch testing in 5.6% of 108 children with atopic dermatitis [74,75]. Banana may also be involved in pollen-food allergy syndrome through profilin-mediated cross-reactivity; profilin sensitization is particularly relevant in pollen-allergic populations, and banana is among the plant foods commonly associated with profilin hypersensitivity [73]. In addition, banana allergy may be associated with birch pollen sensitization through pollen–fruit cross-reactivity and with natural rubber latex through latex–fruit syndrome [72,76].

Six proteins have been identified as major allergens causing banana allergy: Mus a 1 (profilin), Mus a 2 (class I chitinase), Mus a 3 (nsLTP), Mus a 4 (thaumatin-like protein), Mus a 5 (β-1,3-glucanase), and Mus a 6 (ascorbate peroxidase) [77,78]. Mus a 1 (profilin) has been implicated in pollen–fruit cross-reactivity, whereas Mus a 2 (class I chitinase) is associated with latex–fruit syndrome [70,72]. Class I chitinases represented by the 32-kDa and 34-kDa allergens have also been identified as major allergens associated with banana anaphylaxis [79,80].

The clinical spectrum ranges from OAS to urticaria, gastrointestinal symptoms, and potentially life-threatening anaphylaxis. In one study, moderate, severe, and mild reactions accounted for 70%, 23%, and 7% of cases, respectively [79]. Gabrielli et al. reported that banana accounted for 10.8% (27 patients) of fruit-induced anaphylaxis cases in Canada [79]. At the same time, data from the European Food-Induced Anaphylaxis Registry identified banana, together with kiwi, apple, and peach, among the most common fresh fruits implicated in anaphylaxis [80]. Severe anaphylaxis has been associated with reactions occurring during spring and with past or current eczema. Delayed reactions have also been reported, with onset occurring up to 3 h after ingestion and a median onset of approximately 60 min; some cases occurred without identifiable cofactors [81,82,83]. Exercise-induced anaphylaxis following banana ingestion has also been described [84].

Diagnosis may be challenging because commercial banana extracts are not standardized. Prick-to-prick testing has been reported to have greater sensitivity than commercial banana extracts and serum-specific IgE testing [72]. Anaphylaxis despite a negative commercial SPT has also been reported [81]. Therefore, in patients with a convincing clinical history and negative testing with commercial extracts, prick-to-prick testing and, when appropriate, OFC may be required to establish the diagnosis.

### 3.9. Arecaceae

The order Arecales contains a single family, Arecaceae (also known as Palmae), which comprises the palms. Among its edible fruits, dates are particularly popular in Arab and Middle Eastern countries and have been reported to have allergenic potential, including a case of anaphylaxis in an adult [85,86]. Another relevant member of the Arecaceae family is coconut (*Cocos nucifera*), which is widespread in coastal tropical regions. Coconut is botanically a drupe (or stone fruit) rather than a tree nut. Its endosperm contains a substantial amount of liquid, known as coconut water, and the fruit can therefore be consumed both as food and as a beverage. Coconut is also widely used in skin and hair care products, which may represent a potential source of sensitization, particularly in children with atopic dermatitis [87]. Coconut allergy is rare, and its clinical manifestations range from OAS to anaphylaxis.

The edible portion of the fruit consists of approximately 25% albumins and 75% globulins, with the latter including proteins implicated in allergic reactions, such as Coc n 2 (7 S globulin) and Coc n 4 (11 S globulin, cocosin). Sensitization to coconut is relatively common among consumers, although only 50% of patients with a positive SPT had clinically relevant coconut allergy; this proportion increased to 60% among subjects with coconut-specific IgE, according to Kruse et al. [88]. Cosensitization with soy, tree nuts, legumes, and seeds was common [89]. This pattern of cross-reactivity is related to shared 7 S and/or 11 S globulins [90,91].

Anaphylactic reactions to coconut have also been reported, accounting for up to 50% of allergic reactions to this fruit in the study by Kruse et al. [88,91,92,93]. All reported cases in that study had a positive SPT to coconut extract [88].

### 3.10. Lythraceae

Pomegranate (*Punica granatum*) is a fruit-bearing plant belonging to the Lythraceae family. It is commonly consumed in Mediterranean regions, both fresh and in processed forms such as juice. Pomegranate fruits have been reported to contain a 29-kDa protein and a 9–12-kDa protein, the latter of which has been characterized as an LTP [94]. LTPs are also well-characterized allergens in several Rosaceae fruits and may contribute to cross-reactivity among LTP-sensitized patients [94,95]. Several cases of pomegranate allergy have been reported [96]. Clinical manifestations include generalized pruritus, urticaria, and abdominal pain, while anaphylaxis has also been described [96,97]. In the reported anaphylactic cases, prick-by-prick testing with pomegranate and LTP extract was positive [96,97]. Another case of pomegranate-induced anaphylaxis, associated with respiratory symptoms, was reported in a child [98]. Pomegranate has also been implicated in exercise-induced cutaneous allergic reactions, including a case involving a 17-year-old female following ingestion of pomegranate seeds [99].

### 3.11. Rosaceae

The Rosaceae family comprises a wide range of edible fruits, seeds, and berries, as well as ornamental species such as roses and hawthorns [100]. Clinically relevant allergenic species are mainly distributed across three clades: Amygdaleae, Maleae, and Rosoideae. Amygdaleae includes species of the genus *Prunus*, such as almond (*Prunus dulcis*), peach (*P. persica*), apricot (*P. armeniaca*), plum (*P. domestica*), and cherry (*P. avium*, *P. cerasus*). Maleae includes pome fruits such as apple (*Malus*) and pear (*Pyrus*), whereas Rosoideae includes mainly herbaceous or shrubby species such as strawberry (*Fragaria* × *ananassa*) and raspberry (*Rubus idaeus*), which produce aggregate fruits composed of multiple small drupelets [100].

Clinical manifestations of Rosaceae fruit allergy are heterogeneous, ranging from OAS to systemic reactions, including urticaria, asthma, and anaphylaxis. Geographic differences in pollen exposure contribute substantially to the variability in sensitization profiles and clinical phenotypes [101,102]. In birch-endemic regions of Northern and Central Europe, pollen-food allergy syndrome (PFAS) represents the predominant clinical phenotype. Epidemiological studies report that up to 70% of birch pollen-allergic patients develop PFAS involving apple, peach, cherry, apricot, hazelnut, carrot, and pear [103,104,105,106]. In this setting, primary sensitization to the birch pollen allergen Bet v 1 drives cross-reactivity with homologous PR-10 proteins in Rosaceae fruits, including Mal d 1 (apple), Pru p 1 (peach), and Pru ar 1 (apricot). Sensitization to profilins, including Bet v 2, may further broaden cross-reactivity within the family through homologous proteins such as Mal d 4 (apple), Pru p 4 (peach), and Pru ar 4 (apricot) [106,107]. These heat- and digestion-labile proteins are generally associated with predominantly oropharyngeal symptoms [98]. Additional molecular mechanisms contribute to Rosaceae fruit allergy. Phenylcoumaran benzylic ether reductase (Bet v 6) and homologous proteins in plant-derived foods, such as Pyr c 5 in pear, represent another family of cross-reactive allergens with enzymatic activity and clinically relevant IgE binding [106,107]. A German study found IgE reactivity to a Bet v 6 allergen in 12% (9 out of 76) of sera from patients allergic to birch pollen [108]. In contrast, in Mediterranean populations, primary sensitization is predominantly directed against LTPs, including Pru p 3 (peach), Pru ar 3 (apricot), and Mal d 3 (apple) [109]. Unlike PR-10 proteins, nsLTPs are highly resistant to heat and proteolytic digestion and are predominantly localized in the outer layers of fruits, particularly the peel. Their stability allows them to reach the gastrointestinal mucosa in an immunologically intact form and contributes to systemic reactions rather than isolated OAS. Accordingly, avoidance of the relevant raw fruit may be sufficient for patients with predominantly PR-10- or profilin-mediated local reactions, whereas patients sensitized to stable allergens such as nsLTPs or GRPs require a more cautious assessment of the risk associated with both raw and processed fruit [110,111]. Although LTPs from Rosaceae fruits share structural homology and belong to the same allergen family, their allergenic potency and clinical relevance may differ [112]. Gibberellin-regulated proteins (GRPs) represent another class of plant allergens relevant to Rosaceae fruit allergy. These heat- and digestion-stable glycoproteins, which have antimicrobial functions, have been identified in fruits including peach (Pru p 7) and apricot (Pru m 7), as well as in orange (Cit s 7) and pomegranate (Pun g 7). Primary sensitization often occurs through cypress pollen, with cross-reactivity to homologous fruit GRPs. GRP allergy is frequently associated with severe manifestations, including angioedema and laryngeal oedema, and reactions may also be influenced by cofactors such as physical exercise, alcohol intake, or NSAID use [113].

The molecular heterogeneity of Rosaceae fruit allergy has important diagnostic implications. Diagnosis relies on clinical history, SPT, and measurement of specific IgE [101,114]. Conventional extract-based assays are widely used but may be limited by extensive cross-reactivity and by the loss of labile allergens during extract manufacturing. Prick-to-prick testing with fresh fruits or SPT with frozen fruit juices may preserve clinically relevant allergens, particularly heat- and digestion-labile proteins such as PR-10, and can improve diagnostic accuracy compared with standard extracts [114]. However, extract- or fresh-based testing cannot fully replace CRD, which allows molecular sensitization profiles to be characterized, or OFC, which remains the diagnostic gold standard. Functional assays such as the basophil activation test (BAT) are emerging as additional tools for assessing effector cell activation and potentially improving the prediction of reaction severity [101]. Within the Rosaceae family, individual fruits show distinct epidemiological and molecular patterns.

Apple (*Malus domestica*) is among the most extensively studied Rosaceae fruits. Sensitization occurs mainly through birch pollen-related cross-reactivity involving the PR-10 protein Mal d 1 or through primary sensitization to LTP, particularly Mal d 3. In Northern and Central Europe, apple allergy is usually associated with birch pollen sensitization and predominantly manifests as OAS, whereas in Mediterranean regions systemic reactions, including urticaria and anaphylaxis, are more common and are typically associated with LTP sensitization and cross-reactivity with the peach allergen Pru p 3 [115,116,117]. Epidemiological studies confirm that apple is among the most common fruit sensitizers in Europe, with marked geographic variability related to birch pollen exposure [118,119]. In children, the prevalence of probable apple allergy is generally low but heterogeneous across European centres, reflecting regional sensitization patterns [120].

Apricot (*Prunus armeniaca*) allergy is less frequent but clinically relevant within the spectrum of Rosaceae fruit allergy. Its major allergen is the LTP Pru ar 3, which shows strong structural homology with peach Pru p 3 and contributes to cross-reactivity between these fruits [121,122,123,124]. Clinical manifestations range from OAS to systemic reactions and anaphylaxis, sometimes enhanced by cofactors such as exercise or NSAIDs [124].

Pear (*Pyrus communis*) allergy is most commonly associated with PR-10 sensitization. The major allergen Pyr c 1 is homologous to Bet v 1 and shares high sequence similarity with apple Mal d 1, accounting for frequent co-reactivity between apple and pear [125]. Additional allergens include Pyr c 3 (LTP), Pyr c 4 (profilin), and Pyr c 5 (isoflavone reductase), which may contribute to clinical variability [126,127]. Clinically, pear allergy usually presents with mild OAS, although cultivar-dependent differences in allergenicity have been described [128].

Plum (*Prunus domestica* and *Prunus salicina*) is a widely consumed stone fruit with numerous cultivars varying in shape and colour. Although less commonly implicated than peach or apple, plum is clinically relevant because of its close phylogenetic relationship with other *Prunus* species and the molecular similarity of their allergens [129]. The prevalence of plum allergy varies according to population and methodology, with OAS particularly reported among birch pollen-sensitized patients [130,131]. The molecular allergen profile of plum includes four major characterized components. Pru d 1 is a PR-10 protein homologous to birch Bet v 1 and apple Mal d 1; its heat lability and rapid degradation during digestion are consistent with predominantly oral symptoms in birch pollen-sensitized individuals. Pru d 3 is a heat- and digestion-stable nsLTP associated with systemic and severe reactions, particularly in Mediterranean populations. Pru d 4 is a profilin associated with mild OAS and broad cross-reactivity, while Pru d 5 is a thaumatin-like protein (TLP) that has recently been identified as potentially clinically relevant [132]. Systemic plum-induced reactions are uncommon, although both GRP-mediated (Pru d 7) and latex-fruit cross-reactive phenotypes have been documented [133,134].

Peach (*Prunus persica*) is one of the most clinically significant fruit allergens worldwide. In European adults, peach sensitization increased from 5.4% in 2010 to 7.9% in 2013, with peach also representing an important allergen in Japan [135,136]. Its clinical expression varies geographically. In Central and Northern Europe, sensitization is predominantly driven by Pru p 1, a Bet v 1 homolog (PR-10), and mainly results in mild OAS [137]. In Mediterranean regions, primary sensitization occurs predominantly through Pru p 3 (nsLTP), a highly stable 9-kDa protein primarily located in the fruit peel and capable of eliciting systemic reactions, including urticaria, angioedema, and anaphylaxis [138,139,140,141]. Other relevant allergens include Pru p 4 (profilin), which mediates cross-reactivity with pollens and other plant foods [140], and Pru p 7, a GRP strongly associated with severe reactions in patients sensitized to cypress pollen [141,142]. Pru p 9, which is specific to peach tree pollen, is primarily associated with respiratory allergic manifestations [143]. Case reports and cohort studies indicate that peach-induced anaphylaxis may occur despite negative extract-based IgE assays, emphasizing the potential value of CRD [144,145]. In pediatric populations, PR-10 and profilin sensitization is generally associated with mild, localized oral symptoms, whereas LTP-mediated sensitization is associated with systemic reactions and anaphylaxis [146]. Cofactors such as exercise, asthma, or airborne exposure may further exacerbate reaction severity [147]. SLIT targeting Pru p 3 has shown efficacy in increasing tolerated doses, modulating immune responses, and inducing cross-desensitization to other LTP-containing foods, representing a potential disease-modifying intervention for selected LTP-sensitized individuals [148,149].

Nectarine (*Prunus persica*), a glabrous variant of peach, contains allergenic molecules homologous to those of peach and may independently induce systemic reactions, including anaphylaxis [150]. Although documented cases are rare, they highlight distinct immunologic and clinical profiles. A case of systemic anaphylaxis in a 70-year-old man following nectarine ingestion was reported and was considered likely related to cross-reactive sensitization with peach allergens [150]. Latex-fruit syndrome has also been implicated in nectarine-induced reactions, particularly in patients with multiple stone-fruit allergies [134].

Among berries, the strawberry *(Fragaria* × *ananassa*) is one of the most frequently reported fruit allergens in childhood, particularly in self-reported surveys. Population studies conducted in Europe, Asia, and the Middle East have generally reported prevalence rates below 1% among school-age children, although higher estimates have been reported in studies based on parental questionnaires and regional surveys [151,152,153]. Despite these frequent reports, confirmed IgE-mediated strawberry allergy remains relatively uncommon when evaluated by objective testing such as OFC [154,155]. From a molecular perspective, the strawberry contains several allergens, including the Bet v 1-homologous PR-10 protein Fra a 1, profilin Fra a 4, and lipid transfer protein Fra a 3, which contribute to cross-reactivity with birch pollen and other Rosaceae fruits [156].

Other Rosaceae berries, including raspberry (*Rubus idaeus*) and blackberry (*Rubus* spp.), are less frequently implicated but have been associated with IgE-mediated reactions, including rare cases of systemic anaphylaxis. LTPs have been identified as the main allergenic components in several reports, supporting cross-reactivity with other LTP-containing plant foods [157,158,159].

Within the *Prunus* genus, sweet cherry (*Prunus avium*) is another well-characterized allergenic fruit. Its clinical expression varies geographically: in Central and Northern Europe, reactions are mainly related to sensitization to the PR-10 allergen Pru av 1 and typically manifest as OAS, whereas in Mediterranean populations Pru av 3 (LTP) is more frequently involved and may lead to systemic reactions [159,160,161]. Additional allergens, including profilin Pru av 4 and thaumatin-like protein Pru av 2, contribute to the molecular complexity of cherry allergy and to cross-reactivity with pollens and other Rosaceae fruits [162].

Among Rosaceae fruits, peach, apple, and almond are clinically relevant triggers due to their high consumption, allergenic potency, and complex molecular profiles. Their management is particularly challenging because extensive cross-reactivity may complicate the distinction between true clinical allergy and asymptomatic cross-sensitization and does not necessarily translate into clinically relevant cross-allergy. This may result either in unnecessary dietary restrictions or in underestimation of the risk of systemic reactions. Risk assessment and the indication for prescribing adrenaline auto-injectors should therefore consider the molecular sensitization profile, such as LTP- or GRP-mediated sensitization, together with the patient’s clinical history and the presence of cofactors. A personalized, component-resolved approach is therefore important for risk stratification and management. Particular attention is warranted in pediatric patients, in whom LTP sensitization may occur early in life and may be associated with polysensitization to multiple plant-derived foods, particularly Rosaceae fruits and tree nuts. Pediatric cohorts have also reported a substantial burden of systemic reactions and anaphylaxis, emphasizing the importance of careful evaluation of cofactors and molecular risk profiles. Early identification of high-risk sensitization patterns may be particularly relevant in children, who could represent a target population for tolerance-inducing strategies and allergen immunotherapy [163]. Emerging evidence suggests that oral or SLIT, particularly targeting LTPs such as Pru p 3, may increase tolerance and improve quality of life in selected patients, although further studies are needed to define its long-term efficacy and safety [101].

### 3.12. Rutaceae

Rutaceae is a family of flowering plants (order Sapindales), composed of 160 genera and about 2070 species, with a predominantly tropical and subtropical distribution. A number of these plants, notably members of the genus *Citrus*, are important food crops and are commonly consumed worldwide. Numerous edible fruits belong to the genus *Citrus*: bergamot orange (*C. aurantium*), citrange (*C. sinensis* × *Poncirus trifoliata*), clementine (*C. clementina*), kumquat (*C. japonica*), citron (*C. medica*), grapefruit (*C. paradisi*), lemon (*C. limon*), lime (*C. aurantifolia*), orange (*C. sinensis*), shaddock (*C. maxima*), mandarin (*C. reticulata*), and tangerine (*C. tangerina*). Orange is one of the most widely consumed citrus fruits and is commonly included in diets worldwide, both as a fresh fruit and in derived foods and beverages [164]. Despite this, citrus allergy is very uncommon and is often associated with pollinosis and sensitization to other plants due to a phenomenon of cross-reactivity [165]. In fact, pollen may sensitize the respiratory tract, predisposing to allergy to foods containing homologous proteins to those in the sensitizing pollen. The prevalence of sensitization to citrus fruits among children and young adults with a clinical history of pollinosis reaches 39% [166].

The most important orange allergens are Cit s 1, a germin-like protein (GLP) [167]. Cit s 2, a profilin [168], and Cit s 3, an nsLTP [164]. Other allergens have been described more recently, such as Cit s 7 (GRP) [167,169,170]. Cit s 1 and Cit s 2 are the most frequently involved in allergic reactions, while Cit s 3 is considered a minor allergen, with a prevalence of approximately 35% [164]. Regarding cross-reactivity between pollen and orange allergens, Crespo et al. documented a statistically significant correlation between sensitization to Cit s 1 and Cit s 2 and the positive response to SPT, respectively, to cypress (Cit s 1) and Platanus (Cit s 2) in patients with adverse reactions to oranges [165].

Most patients with orange allergy experience OAS, such as itching and swelling of the mouth, throat, and face [168]. However, cases of anaphylaxis from oranges consumed in different forms have been described in the literature. In fact, cases of anaphylaxis following the ingestion of orange juice have been described [171,172], as well as reactions caused by orange seeds [173]. These latter patients reacted to citrus seeds in the absence of clinical reactivity to fruit pulp/peel. Furthermore, cases of orange anaphylaxis have been described in which cofactors, such as physical exercise, played a fundamental role [174,175]. Allergies to other types of fruit belonging to the genus *Citrus* are less common and therefore less studied in the literature. Cases of anaphylaxis caused by lemon seeds [176], kumquat [177], and mandarin [178] have been reported. In these cases, the allergens responsible have not always been identified.

### 3.13. Vitaceae

Vitaceae are a family of flowering plants with 20 genera and around 910 known species, with a predominantly tropical, subtropical, and warm-temperate distribution. The most common genus is *Vitis*, which includes *Vitis vinifera*, from which fresh grapes and grape juice can be obtained. Grape allergy is particularly rare.

The major grape allergens reported in the literature are Vit v 1 (LTP), an endochitinase, and a thaumatin-like protein (TLP). Moreover, allergies to other proteins have been reported, including the GRP [179]. A 28-kDa expansin, a 37.5-kDa polygalacturonase-inhibiting protein, a 39-kDa beta-1,3-glucanase, and a 60-kDa protein were identified as minor grape allergens [180].

Symptoms of grape allergy can range from OAS to hives, angioedema, gastrointestinal symptoms, and anaphylactic reactions. Several cases of severe allergic reactions to grapes have been reported in the literature, both in children and adults [181,182,183]. Vassilopoulou et al. reported that anaphylaxis to grapes occurred 5–120 min after ingestion, and several patients reported more than one episode [181]. In the context of cross-reactivity, most patients with grape allergy are sensitized to other LTP-containing plant foods [181]. Anaphylactic reactions to wine and specific grape varieties have also been reported [181]. The entire population was polyallergic, with extensive sensitization to food, pollen, and other allergens, and grapes were likely responsible for primary sensitization in most patients. Moreover, one patient experienced exercise-induced anaphylaxis after grape consumption. All patients tested positive in the prick test for fresh grapes [180]. The study by Kalogeromitros et al. revealed that the causative agent may vary: wine (28.6%); red grapes (25.7%); stuffed vine leaves (22.9%); raisins (8.6%); white grapes (5.7%); wine vinegar (5.7%); and grape juice (2.9%) [183].

Numerous cofactors may also play a role in determining an anaphylactic reaction to grapes, such as alcohol [184] and physical exercise [179,185]. In one case, an anaphylactic reaction was reported after ingesting wine, even though the patient regularly consumed fresh grapes without any problems [185].

### 3.14. Lauraceae

The Lauraceae family is represented by avocado (*Persea americana*), a widely consumed fruit whose intake has increased globally due to its recognized nutritional benefits [186]. Avocado hypersensitivity is frequently associated with latex–fruit syndrome, with reported cross-reactivity to latex, chestnut, banana, and kiwi, suggesting shared antigenic determinants. However, isolated avocado allergy without latex sensitization has also been described, indicating heterogeneous sensitization patterns [5].

From a molecular perspective, only a few avocado allergens have been identified to date, including Pers a 1 (a class I endochitinase) and Pers a 4 (profilin), as well as other proteins such as β-glucanases and thaumatin-like proteins. Recent evidence has identified endo-1,4-β-glucanase as a potential novel allergen involved in IgE-mediated reactions [186]. Severe avocado-related anaphylaxis is rare [187]. The prevalence and clinical characteristics of avocado allergy in pediatric populations remain poorly defined.

### 3.15. Ebenaceae

Persimmon (*Diospyros kaki*), also known as Sharon fruit, belongs to the Ebenaceae family and is widely consumed in East Asian and Mediterranean countries. Early case reports and small case series highlighted the heterogeneity of persimmon allergy, describing patients presenting with OAS, urticaria, angioedema, and anaphylaxis, often in association with pollen sensitization [188]. Pediatric cases have also been documented, demonstrating that persimmon allergy may occur early in life and may involve different immunological mechanisms, including both pollen-related cross-reactivity and primary LTP-mediated sensitization [189].

Several allergenic proteins have been identified in persimmon. Reported allergens include Dio k 4 (profilin), a 17-kDa protein homologous to Bet v 1, a Bet v 6-related isoflavone reductase, and a pectin methylesterase inhibitor of uncertain clinical significance [190]. More recently, LTPs, a thaumatin-like protein (TLP), and a chitinase have also been described as potential allergens involved in immediate hypersensitivity reactions. These molecular components may contribute both to primary sensitization and to cross-reactivity with pollens and plant-derived foods. In particular, sensitization patterns associated with birch pollen allergens, profilins, cross-reactive carbohydrate determinants (CCDs), and LTP-containing fruits have been reported. Recent studies have further expanded the molecular characterization of persimmon allergy by identifying novel allergens, including a TLP and a chitinase, in patients with immediate systemic reactions who were not sensitized to common plant panallergens [191].

### 3.16. Myricaceae

Chinese bayberry *(Myrica rubra*), belonging to the Myricaceae family, has been only rarely implicated in fruit allergy. A single clinical report described a 40-year-old woman with anaphylaxis and generalized urticaria after ingestion of Chinese bayberry. Skin prick-by-prick testing was strongly positive for fresh bayberry and peach, whereas immunoblotting revealed IgE reactivity to 22-, 45-, and 90-kDa proteins, supporting the presence of novel, as-yet-unidentified allergenic components [192].

### 3.17. Cactaceae

The Cactaceae family includes several edible fruits that are increasingly consumed worldwide, particularly in tropical, subtropical, and Mediterranean regions. Among these, cactus fruit (*Opuntia ficus-indica*) and dragon fruit have attracted allergological interest due to rare but potentially severe hypersensitivity reactions reported in the literature. Although allergy to Cactaceae fruits appears to be uncommon, both local and systemic IgE-mediated reactions, including anaphylaxis, have been reported. Early reports described anaphylaxis after ingestion of cactus fruit. Several affected patients also presented OAS to multiple fruits and vegetables and sensitization to pollen-associated plant panallergens, particularly profilins. Immunoblotting studies identified multiple IgE-reactive bands, including a low-molecular-weight protein compatible with profilin, suggesting that cross-reactivity with pollens may contribute to sensitization. Additional unidentified proteins of higher molecular weight have also been detected and may be implicated in systemic reactions. Possible associations with latex sensitization have also been proposed [193]. Dragon fruit, also known as pitaya, has gained increasing popularity in Europe and North America because of its exotic appearance and use in juices and processed beverages. Several cases of immediate allergic reactions and anaphylaxis after pitaya ingestion have been reported. Molecular investigations have reported IgE reactivity to a low-molecular-weight protein identified as an nsLTP. More recent reports have also documented dragon fruit-induced anaphylaxis in individuals without sensitization to pollen, latex, or other plant-derived foods, indicating that pitaya allergy may occur independently of cross-reactivity with other fruit species [194].

### 3.18. Sapindaceae

Lychee (*Litchi chinensis*) has been reported to cause anaphylaxis. In a reported case, profilin-mediated cross-reactivity was implicated, particularly in a pollen-sensitized patient [195].

## 4. Conclusions

Fresh fruit allergy is a heterogeneous condition characterized by diverse clinical phenotypes, molecular sensitization profiles, and patterns of cross-reactivity. In this review, an established botanical fruit-classification framework was reinterpreted from an allergological perspective to organize the available evidence across 18 botanical families, integrating botanical relationships with molecular allergology and clinical phenotypes. Importantly, botanical relatedness should not be considered a predictor of clinical cross-reactivity, which is primarily determined by shared or homologous allergenic proteins and may occur both within and across botanical families.

From a clinical perspective, diagnosis and management should be individualized according to the patient’s clinical history, molecular sensitization profile, confirmed trigger, and risk of systemic reactions. The stability of the relevant allergenic proteins is an important consideration: heat- and digestion-labile allergens, such as PR-10 proteins and profilins, are generally associated with predominantly local PFAS/OAS, whereas more heat- and digestion-stable allergens, particularly nsLTPs and GRPs, may retain their allergenic activity after heating and may be associated with systemic reactions. Accordingly, peeling or cooking may be tolerated by selected patients with predominantly local PFAS, depending on the relevant allergen profile, but should not be considered universally protective. Epinephrine auto-injectors should be prescribed when clinically indicated according to individual risk. The proposed diagnostic and management pathway is summarized in Figure 1.

The geographical distribution and strength of evidence vary considerably among fruit allergies, with evidence for less frequently reported fruits often limited to small series or isolated case reports. Further prospective and multicenter studies, particularly in children and using standardized diagnostic approaches, are needed to better define the epidemiology, molecular sensitization profiles, and clinical phenotypes of fresh fruit allergy and to evaluate the clinical utility of integrating botanical classification with molecular allergology.

## Figures and Tables

**Table 1:** Botanical classification of fresh fruits with representative fruits, main molecular allergens, and principal cross-reactivity patterns. The classification was adapted from the framework proposed by Pennington [2] and further refined according to the allergological literature reviewed in the present article. Abbreviations: CCDs, cross-reactive carbohydrate determinants; GRP, gibberellin-regulated protein; LTP, lipid transfer protein; nsLTP, nonspecific lipid transfer protein; PR-10, pathogenesis-related protein family 10.

Botanical Family	Representative Fresh Fruits	Main Molecular Allergens	Main Cross-Reactivity Patterns
Actinidiaceae	Kiwifruit (*Actinidia deliciosa*, *Actinidia chinensis*)	Act d 1 (actinidin, cysteine protease); Act d 2 (thaumatin-like protein); Act d 5 (kiwellin); Act d 8 and Act c 8 (PR-10 proteins); Act d 9 (profilin); Act d 10 and Act c 10 (nsLTPs).	Cross-reactivity with birch pollen and other pollens via PR-10 proteins and profilin has been reported. Latex–fruit cross-reactivity, particularly involving homologous class I chitinases, has also been described.
Anacardiaceae	Mango (*Mangifera indica*)	Man i 1 (class IV chitinase); Man i 2 (PR-10 protein); Man i 4 (profilin).	Cross-reactivity with other Anacardiaceae foods, including cashew and pistachio, has been described. Profilin- and lipid transfer protein (LTP)-mediated cross-reactivity with other plant foods may also occur.
Bromeliaceae	Pineapple (*Ananas comosus*)	Ana c 1 (profilin); Ana c 2 (bromelain, cysteine protease).	Pollen–food cross-reactivity mediated by profilin has been reported. Serologic cross-reactivity involving cross-reactive carbohydrate determinants (CCDs) has also been described.
Caricaceae	Papaya (*Carica papaya*)	Car p 1 (papain, cysteine protease); chymopapain (cysteine protease); Cari p 1 (endopolygalacturonase).	Latex–fruit cross-reactivity, particularly involving class II chitinases, has been reported.
Cucurbitaceae	Watermelon (*Citrullus lanatus*); melon (*Cucumis melo*); winter melon *(Benincasa hispida)*	Cuc m 1 (cucumisin, serine protease); Cuc m 2 (profilin); Cuc m 3 (PR-1 protein); nsLTPs	Ragweed and grass pollen–fruit cross-reactivity, particularly involving profilin, has been reported. Profilin-mediated cross-reactivity among Cucurbitaceae fruits and nsLTP-mediated cross-reactivity with other LTP-containing plant foods may also occur.
Ericaceae	Blueberry (*Vaccinium corymbosum)*; cranberry (*Vaccinium macrocarpon*); lingonberry (*Vaccinium vitis-idaea*); bilberry (*Vaccinium myrtillus*); huckleberry (*Gaylussacia baccata)*	LTPs; PR-10 proteins; profilins. Individual allergen components remain incompletely characterized.	Pollen-associated cross-reactivity involving profilin and PR-10 proteins has been reported. LTP-mediated cross-reactivity with other LTP-containing plant foods has also been described, although the available evidence is limited.
Moraceae	Fig (*Ficus carica*); mulberry (*Morus* spp.)	Fic c 4 (profilin); ficin (cysteine protease); fig LTP; PR-10 proteins.	Birch pollen–fruit cross-reactivity involving PR-10-related proteins has been reported. Cross-reactivity between *Ficus benjamina* latex and fig has also been described. Cross-reactivity among Moraceae fruits has been reported.
Musaceae	Banana (*Musa* spp.)	Mus a 1 (profilin); Mus a 2 (class I chitinase); Mus a 3 (nsLTP); Mus a 4 (thaumatin-like protein); Mus a 5 (β-1,3-glucanase); Mus a 6 (ascorbate peroxidase).	Pollen–fruit cross-reactivity involving profilin, particularly with grass and birch pollen, has been reported. Latex–fruit cross-reactivity, particularly involving class I chitinases, has also been described.
Arecaceae	Date (*Phoenix dactylifera*); coconut (*Cocos nucifera*)	Coc n 2 (7 S/vicilin-like globulin); Coc n 4 (11 S/legumin-like globulin); date fruit allergens remain incompletely characterized.	Cross-reactivity between coconut storage proteins and homologous 7 S and 11 S globulins from tree nuts and legumes has been reported.
Lythraceae	Pomegranate (*Punica granatum*)	Pun g 1 (nsLTP); Pun g 7 (gibberellin-regulated protein, GRP); Pun g 14 (class III chitinase).	LTP-mediated cross-reactivity with other LTP-containing plant foods has been reported. GRP-mediated cross-reactivity with other GRP-containing fruits and cypress pollen has also been described.
Rosaceae	Almond (*Prunus dulcis*); peach (*Prunus persica*); apricot (*Prunus armeniaca*); plum (*Prunus domestica*); cherry (*Prunus avium, Prunus cerasus*); apple (*Malus domestica*); pear (*Pyrus communis*); strawberry (*Fragaria* × *ananassa*); raspberry (*Rubus idaeus*); blackberry (*Rubus fruticosus*)	PR-10 proteins (e.g., Mal d 1, Pru p 1, Pru ar 1, Pru av 1, Pyr c 1, Fra a 1, Rub i 1, Pru d 1); profilins (e.g., Mal d 4, Pru p 4, Pru av 4, Pyr c 4, Fra a 4, Pru d 4); nsLTPs (e.g., Mal d 3, Pru p 3, Pru ar 3, Pru d 3, Pru av 3, Pyr c 3, Fra a 3, Rub i 3, Pru d 3); GRPs (Pru p 7, Pru av 7, Fra a GRP); thaumatin-like proteins (e.g., Mal d 2, Pru av 2).	Birch pollen–fruit cross-reactivity involving PR-10 proteins is well characterized. Pollen-associated cross-reactivity involving profilins has also been described. nsLTP-mediated cross-reactivity with other LTP-containing plant foods and within Rosaceae is well recognized. GRP-mediated cross-reactivity with other GRP-containing fruits and Cupressaceae pollen has also been reported.
Rutaceae	Kumquat (*Citrus japonica*); lemon (*Citrus limon*); orange (*Citrus sinensis*); mandarin (*Citrus reticulata*)	Cit s 1 (germin-like protein); Cit s 2 (profilin); Cit s 3 (nsLTP); Cit s 7 (GRP); Cit l 1 (germin-like protein); Cit l 3 and Cit r 3 (nsLTPs).	Pollen-associated cross-reactivity involving profilin and germin-like proteins has been reported. GRP-mediated cross-reactivity with other GRP-containing fruits and cypress pollen has also been described. nsLTP-mediated cross-reactivity with other LTP-containing plant foods may occur.
Vitaceae	Grape (*Vitis vinifera*)	Vit v 1 (nsLTP); Vit v GRP (gibberellin-regulated protein); endochitinase; thaumatin-like protein.	LTP-mediated cross-reactivity with other LTP-containing plant foods has been reported. GRP-mediated cross-reactivity with other GRP-containing foods has also been suggested, although the evidence remains limited.
Lauraceae	Avocado (*Persea americana*)	Pers a 1 (class I chitinase/endochitinase); Pers a 4 (profilin).	Latex–fruit cross-reactivity involving avocado, banana, kiwifruit, and chestnut has been described, particularly in association with homologous class I chitinases.
Ebenaceae	Persimmon (*Diospyros kaki*)	Dio k 4 (profilin); Bet v 1-homologous PR-10-like protein; isoflavone reductase/phenylcoumaran benzylic ether reductase; chitinase; thaumatin-like protein.	Pollen-associated cross-reactivity involving profilin has been reported. Cross-reactivity involving carbohydrate determinants and possible LTP-mediated cross-reactivity with other plant foods have also been described.
Myricaceae	Chinese bayberry (*Morella rubra/Myrica rubra*)	No molecular allergen has been established for the fruit species considered in the reviewed literature.	No established cross-reactivity pattern has been demonstrated. Concomitant sensitization to peach has been reported.
Cactaceae	Cactus fruit (*Opuntia ficus-indica*); dragon fruit *(Selenicereus undatus)*	A profilin-like protein has been reported in cactus fruit; an nsLTP has been identified in dragon fruit.	Pollen-associated cross-reactivity via profilin has been suggested for cactus fruit. nsLTP-mediated cross-reactivity with other LTP-containing plant foods has been reported for dragon fruit, although primary allergy independent of cross-sensitization has also been described.
Sapindaceae	Lychee (*Litchi chinensis*)	Lit c 1 (profilin); Lit c TPI (triose-phosphate isomerase); Lit c IFR (isoflavone reductase); Bet v 6-related phenylcoumaran benzylic ether reductase.	Profilin-mediated pollen–fruit cross-reactivity, particularly involving mugwort pollen, has been reported. Cross-reactivity with other profilin-containing plant foods may also occur.

## Data Availability

No new data were created or analyzed in this study. Data Sharing is not applicable to this article.

## Abbreviations

The following abbreviations are used in this manuscript:

Act d	Actinidia deliciosa allergen component
Ana c	Ananas comosus allergen component
BAT	Basophil Activation Test
CCD	Cross-reactive Carbohydrate Determinants
CI	Confidence Interval
CRD	Component-Resolved Diagnostics
Der p 1	Dermatophagoides pteronyssinus allergen 1
FDEIA	Food-Dependent Exercise-Induced Anaphylaxis
GLP	Germin-Like Protein
GRP	Gibberellin-Regulated Protein
IgE	Immunoglobulin E
LTP	Lipid Transfer Protein
nsLTP	Non-specific Lipid Transfer Protein
NSAIDs	Non-Steroidal Anti-Inflammatory Drugs
OAS	Oral Allergy Syndrome
OFC	Oral Food Challenge
PFAS	Pollen-Food Allergy Syndrome
PR	Pathogenesis-Related Protein
PR-10	Pathogenesis-Related Protein Family 10
PR-14	Pathogenesis-Related Protein Family 14
sIgE	Specific Immunoglobulin E
SLIT	Sublingual Immunotherapy
SPT	Skin Prick Test
TLP	Thaumatin-Like Protein
